# Endodontic Treatment of a Dilacerated Maxillary Second Premolar With a Severely Curved Root Canal: A Case Report and Literature Review

**DOI:** 10.7759/cureus.59590

**Published:** 2024-05-03

**Authors:** He Liu, Jing Hao, Ya Shen

**Affiliations:** 1 Division of Endodontics, Department of Oral Biological and Medical Sciences, University of British Columbia, Vancouver, CAN; 2 Department of Conservative and Endodontic Dentistry, Hangzhou Stomatology Hospital, Hangzhou, CHN; 3 Division of Endodontics, Department of Oral Biological and Medical Sciences, Faculty of Dentistry, University of British Columbia, Vancouver, CAN

**Keywords:** root canal curved canal, cone-beam computed tomography, root canal treatment (rct), maxillary second premolars, endodontic treatment

## Abstract

Dilaceration is a developmental anomaly characterized by a sharp change in the axial inclination between the crown and the root of a tooth. Severe root curvature in a dilacerated tooth can greatly complicate root canal treatment. This case report details the successful endodontic treatment of a dilacerated maxillary second premolar with significant root curvature. It highlights the importance of a thorough understanding of root canal anatomy and demonstrates the effectiveness of using pre-curved hand files along with heat-treated nickel-titanium rotary instruments in navigating complex root structures to achieve successful treatment outcomes.

## Introduction

The success of root canal treatment mainly depends on meticulous cleaning, shaping of the canal to eradicate bacteria, and effective disinfection [1-6]. Equally critical is achieving a thorough seal with appropriate filling materials to prevent future infections and ensure the longevity of the procedure [7-9]. A deep understanding of both common and unusual root canal anatomies is vital for success in these treatments [10].

Root dilaceration, which involves an abnormal bend or distortion in the root or crown of a tooth, typically develops during tooth formation due to trauma, genetic factors, or environmental influences [11]. A dilacerated tooth with severe root curvature can significantly complicate root canal treatment, potentially leading to procedural errors such as canal transportation, ledge formation, blockages, or instrument fracture [12]. These issues can reduce the efficacy of disinfection, compromise the apical seal, and lead to undesirable outcomes.

Dilaceration can affect any tooth, though its prevalence varies [11]. A study by Malcić et al., which analyzed 953 periapical intraoral and 488 panoramic radiographs from Caucasian patients, reported the highest incidence of root dilaceration in mandibular third molars (24.1%), followed by maxillary first molars (15.3%), second molars (11.4%), and third molars (8.1%) [13]. Dilacerations were less common in the mandible than in the maxilla, and particularly rare in maxillary second premolars (6.7%).

Research by Yan et al. on 1,118 CBCT scans of maxillary second premolars in a Western Chinese population found that 94.2% of these teeth are single-rooted and 55.1% have one canal [14]. The most common orientation for curvature in these premolars is mesiodistal (38.8%), with straight (30.9%) and buccopalatal (27.6%) orientations also common, and S-shaped curvatures being relatively rare (2.7%).

This case report details the successful endodontic treatment of a dilacerated maxillary second premolar with significant root curvature, highlighting the importance of precise anatomical knowledge. The strategic use of pre-curved hand files and heat-treated nickel-titanium rotary files proved crucial in navigating complex root structures, demonstrating their effectiveness in achieving optimal treatment outcomes.

## Case presentation

A 17-year-old female Chinese patient presented with moderate pain rated 4 on the visual analog scale in response to thermal stimuli in the upper left posterior teeth, persisting for one week. The pain, which did not worsen with chewing or biting, was not severe enough to disturb her sleep or require the use of antibiotics or painkillers. Two months prior, the patient's maxillary left second premolar had undergone direct pulp capping for a deep caries lesion. Her medical history was unremarkable, and she was classified as American Society of Anesthesiologists (ASA) I, indicating good general health with no systemic diseases. She also maintained good oral hygiene and had no deleterious or parafunctional habits.

Clinical examination showed normal gingiva around tooth #25, no palpation tenderness at the root apex, extensive composite resin restoration on the occlusal and mesial surfaces, normal tooth mobility, and a negative percussion test. Periodontal probing was within normal limits. Cold testing with Endo-Frost cold spray (Coltene, Altstätten, Switzerland) on the maxillary left second premolar and control teeth (mandibular left second premolar, mandibular left first molar, and maxillary right second premolar) revealed a brief, sharp pain in the control teeth, indicative of healthy pulp vitality. In contrast, the maxillary left second premolar responded with prolonged, lingering pain lasting 30 seconds post-stimulation.

**Figure 1:**
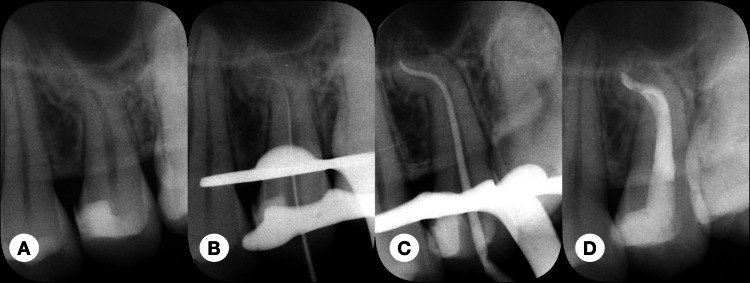
Pre-operative (A), working length confirmation (B), cone-fit (C), and post-obturation (D) radiographs of the maxillary left second premolar

**Figure 2:**
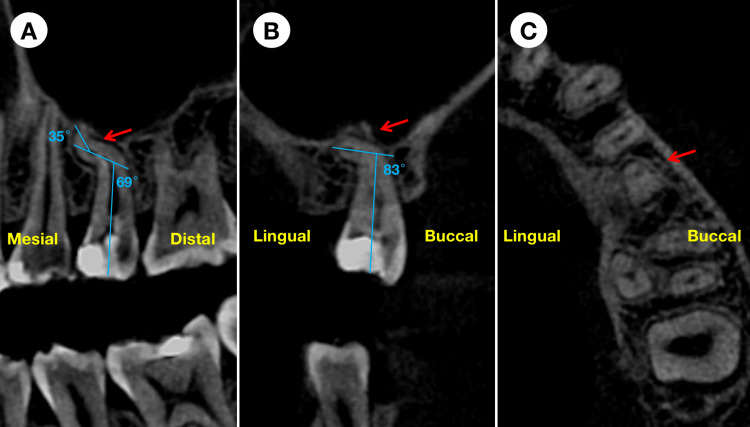
Sagittal (A), coronal (B), and axial (C) sections of cone-beam computed tomography images of the maxillary left second premolar There were multiple root curvatures, specifically mesial curvature in the sagittal section (A) and lingual curvature in the coronal section (B).

A periapical radiograph showed occlusal and mesial decay with potential pulp involvement in tooth #25. The tooth exhibited a severely "S-shaped" root, though no periapical radiolucency was evident (Figure 1A). Limited field-of-view cone-beam computed tomography (CBCT) images confirmed multiple root curvatures, specifically mesial curvature in the sagittal section and lingual curvature in the coronal section (Figure 1). In the sagittal section of the CBCT image, tooth #25 displayed first and second curvatures of 69° and 35°, respectively (Figure 2A). Meanwhile, in the coronal section of the CBCT image, the curvature of tooth #25 was measured at 83° (Figure 2B).

The diagnosis was symptomatic irreversible pulpitis with normal periapical tissue in the maxillary second premolar. The treatment plan included root canal treatment followed by a post-supported full crown restoration for the maxillary second premolar. The patient was informed and consented to the treatment plan and procedures throughout the pre-operative and entire treatment process.

Following the administration of local infiltration anesthesia, a full capsule (1.7 mL) of 4% articaine with 1:100,000 adrenaline (Septanest, Septodont, France) was injected on the buccal side, and a half capsule (0.85 mL) was administered on the palatal side. The tooth was isolated with a rubber dam. Subsequently, composite resin removal and access opening procedures were carried out under a dental operating microscope (M525 F40; Leica, Heerbrugg, Switzerland). Straight-line access was established, and pre-curved #8 and #10 K-files (Dentsply Tulsa Dental, Oklahoma City, USA) were used to negotiate the root canal. The working length was determined using an electronic apex locator, and a periapical radiograph was taken to confirm the working length (Figure 1B). A copious 1% sodium hypochlorite solution (NaOCl) was used to irrigate the canal. Due to the patient's time constraints, root canal treatment was scheduled for the next appointment. The pulp chamber was dressed with calcium hydroxide paste (Pulpdent™ paste; Pulpdent Corporation, Watertown, USA). A sterile cotton pellet was placed in the pulp chamber, and glass ionomer cement was used as a temporary filling material to seal the cavity.

**Figure 3:**
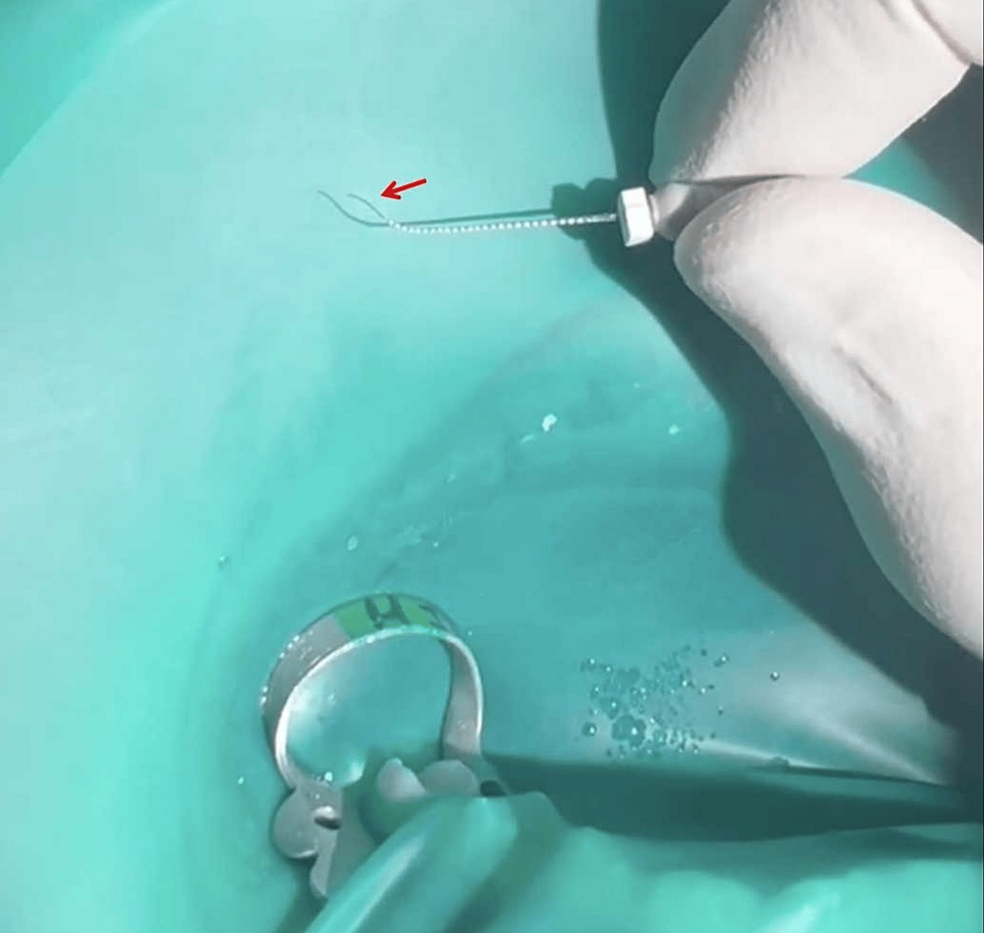
The photo captured under the dental operating microscope shows a pre-curved K-file navigating through the multiple curves (red arrow) of the root

The patient returned one week later and was asymptomatic. The tooth was then isolated with a rubber dam. The temporary filling material and the cotton pellet were removed under the dental operating microscope. A copious amount of 1% NaOCl was used to irrigate the canal to remove the calcium hydroxide paste. Pre-curved #10 and #15 K-type files (Dentsply Tulsa Dental) were used to negotiate the canal (Figure 3). M3 Pro Gold (United Dental, Shanghai, China) NiTi rotary files were used to instrument the canal to #25/.04. Copious 1% NaOCl and 17% EDTA were used to irrigate the canals during instrumentation. Final irrigation procedures were performed using 1% NaOCl and 17% EDTA activated by an ultrasonic file. The canals were dried using absorbent paper points (Gapadent Co Ltd, Tianjin, China). Once these irrigation steps were completed, the canals were thoroughly cleansed with sterile water and subsequently dried using absorbent paper points. A trial fitting of the master gutta-percha cones (Gapadent Co Ltd, Tianjin, China) was then confirmed by a periapical radiograph (Figure 1C). Before placing them, the tips of the gutta-percha cones were dipped in a small amount of AH Plus sealer (Dentsply). The root canal filling was completed using the continuous wave obturation technique. A periapical radiograph was taken later to evaluate the quality of the root canal filling (Figure 1D). The access cavity was temporarily sealed again. The patient was advised to wait one week before proceeding with the post-supported full crown restoration.

## Discussion

A tooth with a straight root and canal is rare, as most teeth exhibit varying degrees of curvature along multiple planes throughout their length [11]. This anatomical complexity can significantly influence the outcomes of root canal treatments. Root canal curvature is a primary intraoperative risk factor associated with complications such as canal transportation, asymmetrical dentine removal, and instrument separation [15]. Research has shown that maintaining the natural curvature and shape of the canal during treatment can lead to higher success rates [16].

To enhance the effectiveness of root canal treatments and reduce potential complications, clinicians must possess a thorough understanding of the tooth's curved anatomy prior to the procedure. This knowledge is crucial for developing a precise treatment plan and selecting appropriate techniques, instruments, and equipment for root canal preparation, irrigation, and filling [15]. Such meticulous planning and execution are vital to avoid issues like canal deviation, ledge formation, root perforation, and instrument fracture, ultimately improving treatment outcomes [17-19].

Root dilaceration arises from a developmental anomaly characterized by a sudden change in the axial inclination between the crown and the root of a tooth [11]. Although the exact causes of root dilaceration remain unclear due to a lack of consensus, the most commonly accepted explanation is mechanical trauma to the deciduous predecessor, which can affect the development of the subsequent permanent tooth [11]. Additional factors that may contribute to root dilaceration include ectopic development of the tooth germ, spatial constraints, interference from anatomic structures such as the cortical bone of the maxillary sinus, the mandibular canal, or the nasal fossa, the presence of adjacent cysts or tumors, and mechanical obstruction of eruption due to an ankylosed primary tooth that fails to resorb [11].

In the present case, the root tip of the dilacerated maxillary second premolar was in contact with the sinus floor, suggesting that the root dilaceration could have been influenced by the cortical bone of the maxillary sinus, potentially altering the path of the epithelial diaphragm during development. The curvature of the dilacerated root followed a mesiodistal direction rather than buccopalatal, possibly due to the maxillary sinus floor exerting a mesial force on the root [14].

Root dilaceration can occur in every tooth type. Many studies reported the wide variety of prevalence of root dilaceration in the maxillary second premolar [13,14,20-24], ranging from 0-9% (Table 1). Periapical radiographs are the most appropriate way to diagnose the presence of root dilaceration [11]. CBCT offers significant advantages for diagnosing root dilaceration, providing three-dimensional, high-resolution images that enhance visualization and accuracy [13]. This detailed imaging facilitates precise assessment and effective treatment planning, helping identify the extent and angle of root curvature.

**Table 1:** Studies reporting the prevalence of root dilaceration in maxillary second premolars CBCT: cone-beam computed tomography, NA: non-applicable

Authors(year)	Type of study	Region	Sample size (n)	Percentage (%)
Malčić et al. (2006) [13]	Periapical radiography	Croatia	270	6.7
Malčić et al. (2006) [13]	Panoramic radiography	Croatia	708	4.1
Nabavizadeh et al. (2013) [20]	Periapical radiography	Iran	440	0
Karadaş et al. (2015) [21]	Panoramic radiography	Turkey	1,356	1.9
ALHumaid et al. (2021) [22]	Digital orthopantomography	Saudi Arabia	NA	9
Yan et al. (2021) [14]	CBCT	China	1,118	2.7
Hasan et al. (2023) [23]	CBCT	Iraq	NA	0
El-Naji et al. (2023) [24]	Panoramic radiography	Jordan	1,704	1.3

Root dilaceration can pose a great challenge to root canal instrumentation. Clinicians should select the appropriate techniques, instruments, and equipment. Heat-treated nickel-titanium rotary instruments offer significant advantages in preparing dilacerated canals due to their enhanced flexibility and reduced risk of fracture. These properties allow for more efficient navigation through complex canal geometries, ensuring thorough cleaning while minimizing procedural errors [25,26].

Given the necessity to reduce file engagement and torsional stress during the instrumentation of dilacerated canals, a novel method called tactile controlled activation (TCA) with heat-treated nickel-titanium rotary instruments was developed by Chaniotis [12]. This technique involves the single-stroke activation of a stationary engine-driven file, which only rotates after it is fully engaged in a patent canal, allowing clinicians to gain tactile feedback of the canal's underlying anatomy before activating the file. The TCA method emphasizes minimal stress on the canal system by using smaller diameter files to navigate curvatures gently, engaging the file only after achieving maximum insertion below the curvature's fulcrum and confirming the curve's anatomy and topography.

## Conclusions

This case report describes the successful treatment of a dilacerated maxillary second premolar with significant root curvature. Root dilaceration, a developmental anomaly characterized by an abnormal bend in the root or crown, complicates these procedures by increasing the likelihood of procedural errors, such as canal transportation and instrument fracture, thereby potentially compromising the treatment outcome. Detailed anatomical knowledge and the use of CBCT and heat-treated nickel-titanium rotary files are crucial for accurately identifying and addressing such complexities in root canal anatomy.
